# Somatic Embryogenesis and Plant Regeneration From Primordial Shoot Explants of *Picea abies* (L.) H. Karst. Somatic Trees

**DOI:** 10.3389/fpls.2018.01551

**Published:** 2018-10-24

**Authors:** Saila Varis, Krystyna Klimaszewska, Tuija Aronen

**Affiliations:** ^1^Natural Resources Institute Finland (Luke), Savonlinna, Finland; ^2^Natural Resources Canada, Canadian Forest Service, Laurentian Forestry Centre, Quebec, QC, Canada

**Keywords:** clonal trees, conifer, Norway spruce, recalcitrance, shoot buds

## Abstract

The recalcitrance of adult conifer tissues has prevented vegetative propagation of trees with known and desired characteristics. Somatic embryogenesis (SE) initiation protocol, recently developed for white spruce (*Picea glauca*, Klimaszewska et al., 2011), was applied in order to examine the feasibility, frequency and timing of SE induction from primordial shoots (PS) of Norway spruce *(P. abies*). In total, 39 genotypes were screened from 2015 to 2017 using 4–6 years old trees of SE origin as explant donors. Two genotypes responded: 11Pa3794 produced six proliferating embryonal mass (EM) sublines and 11Pa4066 produced 23 EM sublines. SE initiations occurred at the beginning of April, when the temperature sum (d.d.) started to accumulate, and at the end of October or beginning of November when the chilling unit (ch.u.) sum was over 500. EM sublines from both genotypes contained numerous early somatic embryos as detected by acetocarmine staining. The sublines of 11Pa4066 produced the mean of 78.6 ± 12.8 cotyledonary somatic embryos /g FW, but 11Pa3794 produced only a few cotyledonary somatic embryos that were able to germinate. The original EM lines (from which the trees were regenerated) had produced the same number of somatic embryos in 2011 maturations, which was approximately 120 somatic embryos /g FW. Microsatellite analyses conducted with both responsive genotypes confirmed the genetic stability of the EM sublines compared with the donor trees growing in the field. SE protocol developed for white spruce PS explants was also suitable for PS of Norway spruce if the explants were in the responsive developmental stage.

## Introduction

Vegetative, i.e., asexual propagation enables production of plants of uniform quality and with known, selected characters. Somatic embryogenesis (SE) has become the method of choice for vegetative propagation of conifers (Sutton, 2002) due to its high multiplication rate and the maintenance of juvenility of cell lines via cryopreservation. Of the Nordic conifers, SE is currently the most developed in Norway (*Picea abies* (L.) Karst.) and white spruce (*P. glauca* (Moench) Voss) (Lelu-Walter et al., 2013; Adams et al., 2016; Högberg and Varis, 2016). In the case of Norway spruce, development of cost-efficient vegetative propagation techniques is especially important, since there is shortage of high-quality, bred forest regeneration materials due to irregular flowering of the species, as well as pest and pathogen problems hindering seed production in the seed orchards.

The major drawback of the current SE methodology has been that embryogenic cultures can only be initiated from juvenile plant explants, in practice from zygotic embryos, meaning that mature trees with known characteristics cannot be propagated via SE (Bonga et al., 2010). Initiation of SE from mature conifers would provide a shortcut to production of planting material from selected trees with known and desirable traits. In combination with cost-efficient mass propagation, this would have an enormous impact on forestry with subsequently increased productivity and/or production of tailored raw materials for special end-uses and also for landscaping and Christmas tree production.

To study the recalcitrance problem, Bonga et al. (2010) suggested the use of explants taken from mature trees of somatic embryo origin which potentially could be more responsive in tissue culture. This approach was followed with white spruce (*P. glauca* (Moench) Voss) whereby primordial shoots (PS) excised from vegetative buds of 10-year-old (in 2010) and 17-year-old (in 2017) somatic trees produced somatic embryos that converted to plants (Klimaszewska et al., 2011; Klimaszewska and Rutledge, 2016, and Klimaszewska et al., unpublished). Some donor trees in the above study had gone through a phase change from juvenile to mature, i.e., flowering phase and still responded to SE induction treatment.

The developmental stage of an explant is critical for the outcome of *in vitro* propagation, and often the time window for the positive response has been short (Selby and Harvey, 1985; Monteuuis et al., 2010; Bonga, 2017). In white spruce, positive response for SE inductions from PS was obtained both in the spring and late summer / early autumn, and the best response was from inductions made in late April to early May (Klimaszewska et al., 2011). Bonga (2017) suggested that during switches in the developmental program, like in the vegetative buds from dormancy to bud break, tissues may be more active morphogenetically and acquire the propensity for SE.

Dormancy has been divided to different physiological phases: after growth cessation, the buds enter endodormancy, which changes to ecodormancy under the influence of chilling temperatures during the autumn (Lang et al., 1987). During ecodormancy, the shoot buds are capable of development, but low temperatures during the winter hinder their growth onset until temperature rises and days get longer (Sutinen et al., 2012 and references therein). In Norway spruce, defining the internal developmental stages of the PS is impossible by observing the shoot buds externally, thus the staging has been made based on comparisons of longitudinally cut buds and the temperature (d.d.) and chilling unit (ch.u.) sums (Sutinen et al., 2012; Viherä-Aarnio et al., 2014). Also the photoperiod plays a role in the regulation of growth cessation and shoot bud set phenology.

The aim of this study was to examine the feasibility and frequency of SE induction from PS of Norway spruce. We used four to six years old trees of SE origin as donors, and tested explants from clonal trees of 39 genotypes in 2015–2018. The best timing for explant collection was determined by comparing the positive SE initiation results with temperature data. The genetic stability of embryonal mass (EM) sublines was examined with microsatellite markers.

## Materials and Methods

### Donor Trees

**FIGURE 1:**
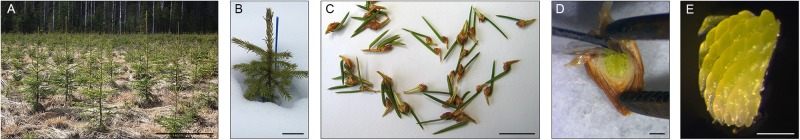
Bud collection from somatic Norway spruce trees and primordial shoot (PS) excision. **(A)** Norway spruce somatic trees growing in an experimental plantation at Punkaharju, Finland in the spring 2018. **(B)** Four years old somatic tree at the first shoot bud collection on April 1, 2016. **(C)** Shoot buds before removal of the outermost scales and disinfection. **(D)** Shoot bud cut longitudinally. **(E)** Quarters of the PS before being placed on the culture medium. Bars: **(A)** = 1 m, **(B)** = 50 mm, **(C)** = 10 mm, **(D)** = 1 mm, **(E)** = 0.5 mm.

Norway spruce SE trees growing in the experimental plantation, at Punkaharju, Finland (61°48′09′′N, 029°18′58′′E) were chosen for the vegetative bud collections (Figures 1A,B). The trees were produced from EMs initiated in 2011 using immature or mature zygotic embryos originating from full-sib seed families between superior trees within Finnish Tree Breeding Program. The crosses were made in a grafted seed orchard situated in southern Finland, and the genotypes also originated from southern Finland. Production of the SE trees was conducted by applying the methods developed by Klimaszewska et al. (2001) and Lelu-Walter et al. (2008), as described in Varis et al. (2017). Maturations of somatic embryos were performed several times between October 2011 and February 2012. Germinations of the cotyledonary somatic embryos were carried out in December 2011 until May 2012. The plantation of the somatic trees was established on a fertile, mounded forest site in May and June 2014 with 280 genotypes and 10 to 40 clonal trees per each genotype. No fertilization was applied to somatic trees under field conditions. Mechanical weeding of the plantation was performed annually.

### Shoot Bud Collections, Explant Preparation and SE Initiations

Shoot bud collection and SE induction protocol from PS explants were the same as developed for white spruce (Klimaszewska et al., 2011). Lateral buds (Figure 1C) were removed mainly from branches of the upper part of the trees where buds were well developed, abundant and not covered by snow and ice, but occasionally also from branches of the lower part. At every collection time, a minimum of 40 lateral buds were collected per genotype from several clonal trees, and in total 39 genotypes were tested (Table 1). Following collection, the buds were stored in plastic tubes at + 4°C for 0 to 4 days before being used in the experiments.

**Table 1:** Norway spruce shoot bud collection dates, number of genotypes tested, responding genotypes and the number of EM sublines.

Collection dates	Number of tested genotypes, new/repeated	Responding genotypes	Responding PS	EM sublines proliferating	EM sublines forming mature SE	SE / g FW	EM sublines forming plants
19 – 29 March 2015	9/0	0					
12 April 2015	4/0	11Pa3794	1	1	1	1.6 (11.2^∗^)	0
17 – 22 April 2015	12/0	0					
1 April 2016	3/0	0					
8 April 2016	3/0	11Pa4066	6	17	16	10.7 – 171.3	9
19 – 27 April 2016	3/1	0					
25 October 2016	1/1	11Pa4066	4	6	6	39.9 – 142.5	6
11 April 2017	2/0	0					
18 April 2017	2/2	11Pa3794	3	2	1	2.2	0
7 November 2017	0/1	11Pa3794	5	2	1	2.1 (15.9^∗^)	^∗∗^

*^∗^EM grown with nurse tissue; ^∗∗^Plants are still being acclimatized. SE / g FW represents range among sublines.*

In the spring 2015, buds were collected from 25 genotypes between March 19 and April 21 (Table 1). In the spring 2016, collection started on April 1 and the last collection was done on April 27. Nine new genotypes were introduced and one responsive genotype (11Pa3794) from previous year was collected for the second time. In the fall 2016, one genotype (11Pa4066) that had been responsive in the spring was collected again on October 25, and also one new genotype was collected on the same day. Buds of 11Pa4066 were collected from four groups of clonal trees: (1) from the same 10 trees as in the spring, (2) from 10 trees growing in a different part of the plantation: (a) five trees from the extreme right row and (b) five trees from the extreme left row, and (3) from one tree growing at the edge of the plantation which was a border tree.

In 2017, buds were collected on April 11 and 18 from four new genotypes and from 11Pa3794 on April 18 and November 8. In the latter collection, the buds of 11Pa3794 were taken from clonal trees growing in two different rows: 40 buds from five trees from each the right and left row.

Before the disinfection of the shoot buds, the outermost scales were removed and they were placed in a 50 ml centrifuge tube, up to 25–30 buds per tube. The disinfection started by shaking the buds in 94% ethanol for 1 min followed by washing in tap water with small amount of Tween-20 for 6 min and then by shaking in 70% ethanol for 2 min, followed by shaking in 10% (v/v) hydrogen peroxide for 8 min. After rinsing three times in sterile water, the buds were placed in a Petri dish on a filter paper moistened with 100 mg l^-1^ polyvinylpyrrolidone (PVP) solution. Buds were cut lengthwise, the two parts of PS were excised and, depending on their size, they were cut lengthwise again into two or more parts as described in Klimaszewska et al., 2011 (Figures 1D,E). The sections of the PS were placed on the surface of the semi-solid MLV medium (Litvay et al., 1985, modified as in Klimaszewska et al., 2001). There were sections of four PS cultured in one Petri dish (90 mm × 15 mm). The medium was supplemented with 9.5 μM 2,4-dichlorophenoxyacetic acid (2,4-D) and 4.5 μM 6-benzyladenine (BA).

**FIGURE 2:**
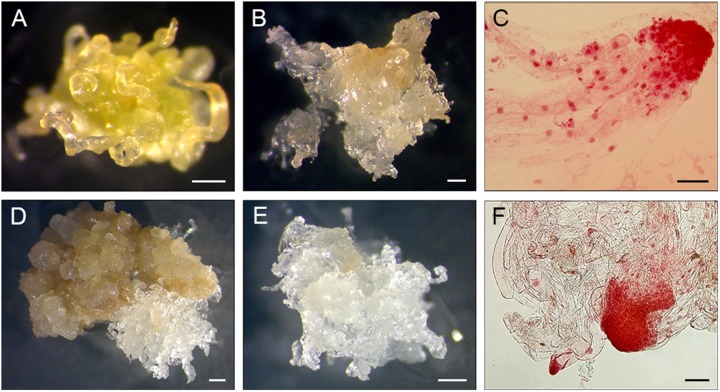
Induction of EM sublines from PS explants. **(A-C)** 11Pa3794 genotype, **(D–F)** 11Pa4066 genotype. **(A)** Elongating primordial needles after 21 days of culture. **(B)** Proliferating EM after 8 weeks of culture. **(C)** Early somatic embryos stained with acetocarmine. **(D,E)** Proliferating EM after 6 weeks of culture. **(F)** Early somatic embryos stained with acetocarmine. Bars: **(A,B,D,E)** = 0.5 mm, (**C,F**) = 100 μm.

The cultures were placed at 24°C in darkness and were inspected once a week for contaminations and/or initiations of EM until the explants and calli became necrotic. When the growth of EM was clearly visible it was subcultured onto fresh medium separately from each section of a PS and considered a subline (Figures 2A,B,D,E). EM were subcultured at 14-day intervals.

### Microscopic Observation, Nurse Tissue Co-culture Experiment, Maturation of Early Somatic Embryos and Conversion to Plants

To detect whether SE was initiated, samples of induced tissues were stained with acetocarmine according to Gupta and Durzan (1987) and observed under the microscope (Figures 2C,F).

**FIGURE 3:**
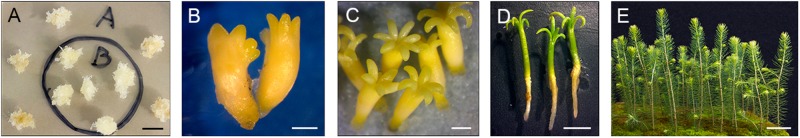
**(A)** Co-culture of EM of 11Pa3794 genotype (**A**, outside circle) and EM 15Pa1029 nurse tissue (**B**, inside circle). **(B,C)** Cotyledonary somatic embryos of 11Pa3794 genotype from different maturation events before and after co-culture with nurse tissue. **(D)** Germinated somatic embryos from subline of 11Pa3794. **(E)** Somatic trees of genotype 11Pa4066 after second growing season. Bars: **(A)** = 1 m, **(B)** = 50 mm, **(C)** = 10 mm, **(D)** = 1 mm, **(E)** = 50 mm.

To enhance somatic embryo production, the EM subline of 11Pa3794 (induced in 2015) was used in the experiment with the nurse embryogenic tissue (Westcott, 1994). The nurse tissue was of 14Pa4623 genotype induced from a zygotic embryo, which consistently produced large numbers of mature somatic embryos. Nurse tissue clumps were positioned either at the circumference of the Petri dish (90 mm× 15 mm, containing 20 ml of MLV proliferation medium) surrounding the clumps of 11Pa3794 placed in the center of a Petri dish or *vice versa*. There were eight to ten clumps at the circumference and five to seven clumps in the center, respectively. Nurse tissue experiment lasted 4 weeks until the subline was matured in November 2015. Similarly, two other EM sublines, induced in November 2017, were co-cultured with nurse tissue of 15Pa1029 (Figure 3A).

The maturation of somatic embryos and conversion to plants were carried out according to a slightly modified protocol that was developed for zygotic embryo SE. EM sublines induced in autumn 2016 and later were matured with reduced concentration of abscisic acid (ABA), 30 μM, instead of previously used 60 μM (Tikkinen et al., 2018a). Also, the *in vitro* germination was shortened to 1 or 2 weeks, and the acclimatization was done in small containers (Tikkinen et al., 2018b).

### Microsatellite Analysis

Genetic stability was examined in the pooled EM sublines if induced from various sections of a single PS or from a single EM subline when it was the only one induced. Microsatellite analyses were conducted from the sublines of 11Pa4066 induced in 2016 and 11Pa3794 induced in 2015. The genomic DNA was extracted from the shoot buds of donor trees and from the EM sublines using either 500 mg samples and the method developed by Lodhi et al. (1994), or 150 mg samples and the method developed by Doyle and Doyle (1990). Microsatellite loci, SpAGD1 (Pfeiffer et al., 1997), WS0022.B15, WS00111.K13, WS0016.O09, and WS0092.A19 (Rungis et al., 2004) were selected for PCR amplification by PTC-100 thermal cycler (MJ Research^®^,QC, Canada). Amplification products were subjected to the electrophoresis in ABI 3500xL (Applied Biosystems) automated sequencer and the genotypes were identified using GeneMapper software (Applied Biosystems).

### Temperature Data

For temperature calculations, data from Finnish Meteorological Institute measurement site situated 1 km away from the experimental SE plantation were used. The temperature sums (d.d.) were calculated by summing the daily mean temperatures exceeding the threshold value 0°C, which was closely correlated with the development of PS in the spring (Sutinen et al., 2012). Chilling unit sums (ch.u., Sarvas, 1974) were calculated based on hourly temperature measurements using 3.5°C as a threshold value (Supplementary Figure S1). Chilling unit sums were used to evaluate PS development in the autumn when d.d. sums stopped to accumulate.

## Results

In 2015, one PS of 11Pa3794 genotype initiated EM (Table 1 and Figures 2A–C). Shoot buds were collected on April 12 and dissected 3 days later (Table 1). The mean temperature on the collection day was 5.7°C and the d.d. sum was 91.5 (Supplementary Figure S1). In 2016, different clonal trees of 11Pa3794 were used for the bud collection on April 22, when d.d. was 108.9, however, the response was negative.

From the spring 2016 collections, six PS of 11Pa4066 responded positively (Figures 2D–F and Table 1) and in total 17 EM sublines were established in culture. The responding buds were collected on April 8, when d.d. sum was 63.1. This genotype responded positively also in the autumn, when one bud from the same clonal tree group, as in the spring, and three buds from the new group of clonal trees (2b) produced six EM sublines. The autumn collection was done on October 25 when the ch.u. sum was 537.6. In the spring positively responding buds were stored for 3 days at + 4°C, and in the autumn either for 1 or 2 days.

In 2017, three buds of 11Pa3794 from the spring collection (51.6 d.d.) and five buds from autumn collection (632.2 ch.u.) responded and in total 17 EM sublines were initiated (Table 1). However, the proliferation of 13 EM sublines was poor and they were discarded after 3 months of culture.

The EM of sublines of 11Pa3794 were translucent / white at the beginning of the initiation, but after 2–4 weeks it changed to opaque, hard and wet. The subline induced from 11Pa3794 in 2015 produced low number or none of good quality somatic embryos (Figure 3B), even though the early somatic embryos were identified in the stained tissue samples (Figure 2C). The mean somatic embryo number was 1.6 per g FW before the co-culture experiment with nurse tissue, and 11.2 per g FW after being proliferated with nurse tissue (Figures 3A,C). All the germinated somatic embryos from the sublines induced in 2015 died during acclimatization even though they formed visible roots and shoots (Figure 3D). Sublines induced in 2017 produced mature embryos when co-cultured for 2 months with nurse tissue, the viability of embryos will be tested in an ongoing study.

The EM sublines of 11Pa4066 were translucent and spiky and produced the mean of 78.6 ± 12.8 viable somatic embryos per g FW. Somatic seedlings were established in a greenhouse (Figure 3E).

Microsatellite analyses were conducted with both responsive genotypes and confirmed the genetic stability of EM sublines when compared with the donor trees growing in the field. The microsatellite loci detected in 11Pa3794 genotype were 188/188 in SpAGD1, 180/234 in WS0022.B15, 226/248 in WS00111.K13, 398/398 in WS0016.O09, and 219/227 in WS0092.A19, and in 11Pa4066 genotype they were 154/166, 178/200, 258/258, 398/404, and 215/223, respectively.

## Discussion

The PS explants and the experimental protocol developed for white spruce was applied to Norway spruce somatic trees as described by Klimaszewska et al. (2011). Five percent of the tested Norway spruce genotypes responded and induced EM. As suggested by Klimaszewska et al. (2011) it is necessary to screen a sufficient number of genotypes to identify the ones with the ability for SE in order to study biochemical and molecular bases of the recalcitrance of mature tree tissues to the SE induction. Moreover, even though we pooled the buds from a group of clonal trees, we concluded that not all of the clonal trees of responding genotypes produced PS that were responsive to SE, similarly to white spruce (Klimaszewska and Rutledge, 2016; Rutledge et al., 2017). The causes of the differential behavior in tissue culture among genotypes and clonal trees within a genotype are not understood but epigenetically heritable mechanisms could be implicated. D’Urso and Brickner (2014) and references therein) and He and Li (2018) and references therein) suggested that previously repressed genes are frequently predisposed for re-activation, a phenomenon called transcriptional or environmental memory. This mechanism requires changes in chromatin structure and a physical interaction with nuclear pore proteins. Such a mechanism allows cells to rapidly mobilize a transcriptional response to an environmental stimuli that they have previously experienced. This could, at least theoretically, explain the higher propensity of somatic tree-derived explants to undergo SE. There have been, however, marked differences in the responses of several pine species compared with spruces, the former being non- or partially responsive to induction of SE when similar experimental approaches were applied (García-Mendiguren et al., 2015; Trontin et al., 2016).

The positive response of the group of clonal trees of two Norway spruce genotypes was repeatable in consecutive years until the end of this study when the somatic trees were 6 years old. We speculate that based on the results with white spruce (Klimaszewska and Rutledge, 2016), this responsiveness will remain for the next several years. The latest experiment with white spruce was carried out in the autumn 2017 and the PS explant of responding 17 years-old somatic trees initiated EM and the mature somatic embryos converted to plants (Klimaszewska et al., unpublished).

The positive responses of Norway spruce PS explants were also repeatable between the seasons; spring and autumn. To fully understand the influence of the PS developmental stage on the initiation of SE, the shoot buds should be collected from the beginning of the bud formation in the late summer through the fall and winter until the full bud break in the spring. To perform an experiment of this magnitude, the trees should be big enough for collection of hundreds of shoot buds, which was not possible in this study due to the small size of the donor trees. However, our study and the results of Klimaszewska et al. (2011) seem to support the suggestion of Bonga (2017) that during switches in the developmental stages tissues may be more capable of initiating SE. Such a switch may occur in late November when the release of endodormancy happens. The timing of the release is dependent on the genotype and environmental conditions, but at least a partial release starts when ch.u. sum exceeds 500 and the full release occurs when ch.u. sum reaches 800–850 (Viherä-Aarnio et al., 2014; Partanen et al., 2016).

The molecular regulation of bud dormancy includes hormonal signaling and specific gene expression, but recent findings pay attention to epigenetic regulation involving modifications of histones, DNA methylation and the synthesis of small non-coding RNAs (D’Urso and Brickner, 2014; Rios et al., 2014). The relationships between genomic DNA cytosine methylation, histone H4 acetylation and bud dormancy was described in *Castanea sativa* (Santamaría et al., 2009), and Valledor et al. (2010) used similar approach to study needle maturation of *Pinus radiata*. In Norway spruce, temperature conditions during zygotic or SE affects the expression of specific genes and thus timing of dehardening and bud burst in the spring, cessation of leader shoot growth in the summer, as well as bud set and cold acclimation in the autumn (Yakovlev et al., 2014; Carneros et al., 2017). Transcription analysis of the bud-burst related genes and other molecular markers like chromatin status may give more insight into timing of the PS explants response to SE.

In the spring, initiation of the PS growth is dependent on the inverse relationship between accumulations of ch.u. and d.d. sums: the higher accumulated ch.u. sum before bud break, the lower d.d. sum is required to initiate PS growth (Viherä-Aarnio et al., 2014). Before the bud break is visible externally, the internal morphological development begins simultaneously with the accumulation of d.d. sum (Sutinen et al., 2012). Primordial needles elongate and cover the shoot apex, the whole PS elongates and the bud scales start to open. In our study, the first signs of needles and shoot elongation were visible only after the PS was cut longitudinally. When the explant was placed on the medium, the primordial needles continued to elongate (Figure 3) but became curved. However, round protuberances (nodules) were not observed on the needle primordia as was reported for white spruce. Such protuberances were often observed to develop into EM (Klimaszewska et al., 2011; Klimaszewska and Rutledge, 2016). In our study with Norway spruce, the growth of EM appeared from the contact area between explant and medium, and thus the actual origin of the EM was not established. A destructive sampling will be necessary to determine the origin of EM in Norway spruce. Apparently, storing the buds at + 4°C for 3 days, as was done with the three batches of buds of 11Pa4066 in 2016, did not affect the result of the experiment compared with fresh buds.

Despite similar embryo productivity, i.e., around 120 embryos per g FW in the original EM lines (derived from the zygotic embryos), the EM sublines of two responsive Norway spruce genotypes grew very differently after initiation. This may be due to differentially expressed proteins like in secondary and tertiary lines of Douglas-fir (Gautier et al., 2018). The EM sublines of 11Pa3794 had much lower embryo productivity than sublines of 11Pa4066, possibly due to the untimely subculture, or other factors. The acetocarmine staining revealed similar early embryos in proliferating sublines of both genotypes, but 11Pa4066 produced more cotyledonary embryos and plants. The initiation of SE-like translucent tissue and the presence of embryo-like structures do not always result in the production of somatic embryos and plants, which was the case with different pine species (Trontin et al., 2016). The change in the appearance of EM and decrease in the somatic embryo maturation yield are usually noticeable after EMs had been subcultured continuously for several months.

Overall, it can be argued that if trees of SE origin provide explants that are more susceptible to SE initiation than similar explants from trees of zygotic origin, they are not completely true-to–type, and it can question if they differ from a seedling in other traits as well. Högberg (2003) studied potential connections between SE success and economically important traits in Norway spruce, and found no adverse correlations. The on-going field experiments with SE trees will provide more detailed information on this subject.

In this study explants only from SE trees were used, and it is not possible to know how their zygotic counterparts would have responded. Even though the somatic seedlings of sublines from Pa3794 did not survive, they produced mature somatic embryos capable of germination. The loss of somatic seedlings during acclimatization happens also in the SE lines of zygotic embryo origin (Tikkinen et al., 2018b), therefore, the lack of survival of Pa4066 somatic seedlings was not unique.

In the sublines of 11Pa3794 initiated in 2015, proliferation in the presence of nurse tissue enhanced the number of mature somatic embryos slightly. In another study with *P. abies*, co-culture with the embryogenic nurse tissue has been used to stimulate proliferation of embryogenic tissue induced in needle explants (Westcott, 1994). Other examples of the successful co-culture with nurse tissue include the enhanced recovery of cryopreserved EM of *Pinus radiata* (Hargreaves et al., 2002) and facilitation of SE initiation from immature zygotic hybrid pine embryos (Hargreaves et al., 2017). The nurse culture is believed to modify culture environment into more favorable one, e.g., by metabolizing sucrose and by excreting growth hormones into the culture medium. The effect of co-culture with nurse tissue on maturation and somatic embryo production has, however, not been reported thus far.

## Conclusion

In conclusion, initiation of SE from PS explants in Norway spruce was possible with certain genotypes. The clonal trees of these genotypes responded repeatedly over the last three years. Further research is needed to learn how SE induction is regulated since there is variation among the clonal trees in their response. The different responsiveness of clonal trees within one genotype provides an opportunity to use advanced gene expression analysis and compare different trees without a confounding genotypic effect (Rutledge et al., 2017). In addition, other explants could be tested such as needle explants, as suggested by Bonga (2017), which also produced SE albeit at limited frequency (Ruaud et al., 1992; Harvengt et al., 2001). Furthermore, the growth of somatic seedlings produced from PS explants will be followed, as well as continuation of SE induction in the responding genotypes.

## Author Contributions

SV designed and carried out the experiments. SV conceived the manuscript, SV, KK, and TA read, wrote and approved the manuscript.

## Conflict of Interest Statement

The authors declare that the research was conducted in the absence of any commercial or financial relationships that could be construed as a potential conflict of interest. The reviewer JMB declared a shared affiliation, though no other collaboration, with one of the authors KK to the handling Editor.
