# Supplementary material for: Somatic Embryogenesis and Plant Regeneration From Primordial Shoot Explants of Picea abies (L.) H. Karst. Somatic Trees
Source: Front Plant Sci. 2018 Oct 24;9:1551. doi: 10.3389/fpls.2018.01551 (PMC6207908; doi:10.3389/fpls.2018.01551)
Supplement: Supplementary file 1 [file Image_1.pdf]

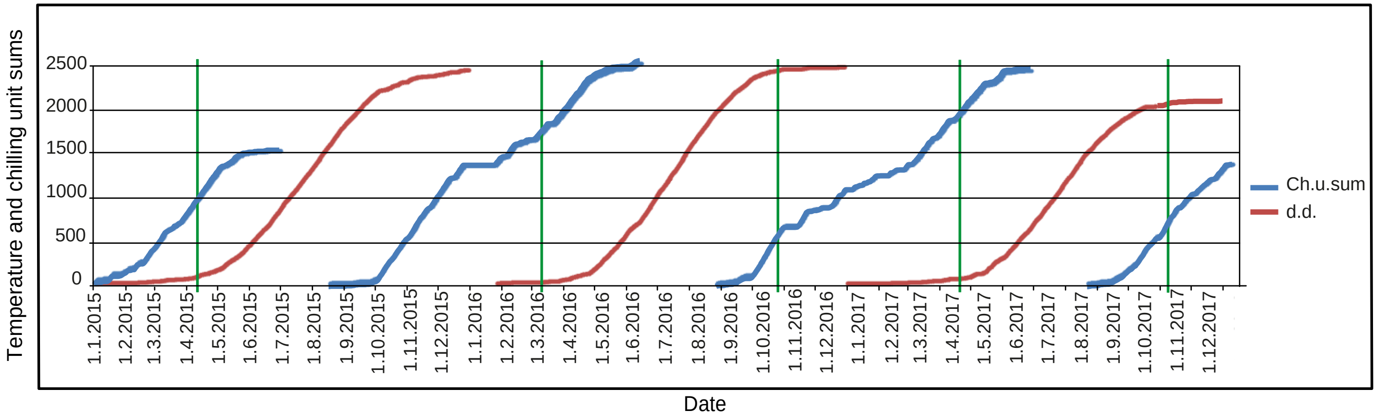

Supplementary Figure S1. Temperature (d.d.) and chilling unit (Ch.u.) sums. Vertical lines show the collection dates of shoot buds with positive response.
